# The Dual Role of TGF**β** in Human Cancer: From Tumor Suppression to Cancer Metastasis

**DOI:** 10.5402/2012/381428

**Published:** 2012-12-24

**Authors:** Jean-Jacques Lebrun

**Affiliations:** Division of Medical Oncology, Department of Medicine, Royal Victoria Hospital, McGill University Health Center, Montreal, QC, Canada H3A 1A1

## Abstract

The transforming growth factor-beta (TGF*β*) superfamily encompasses widespread and evolutionarily conserved polypeptide growth factors that regulate and orchestrate growth and differentiation in all cell types and tissues. While they regulate asymmetric cell division and cell fate determination during early development and embryogenesis, TGF*β* family members play a major regulatory role in hormonal and immune responses, cell growth, cell death and cell immortalization, bone formation, tissue remodeling and repair, and erythropoiesis throughout adult life. The biological and physiological functions of TGF*β*, the founding member of this family, and its receptors are of central importance to human diseases, particularly cancer. By regulating cell growth, death, and immortalization, TGF*β* signaling pathways exert tumor suppressor effects in normal cells and early carcinomas. Thus, it is not surprising that a high number of human tumors arise due to mutations or deletions in the genes coding for the various TGF*β* signaling components. As tumors develop and progress, these protective and cytostatic effects of TGF*β* are often lost. TGF*β* signaling then switches to promote cancer progression, invasion, and tumor metastasis. The molecular mechanisms underlying this dual role of TGF*β* in human cancer will be discussed in depth in this paper, and it will highlight the challenge and importance of developing novel therapeutic strategies specifically aimed at blocking the prometastatic arm of the TGF*β* signaling pathway without affecting its tumor suppressive effects.

## 1. Introduction

The transforming growth factor-beta (TGF*β*) was discovered more than two decades ago and was isolated as a secreted factor from sarcoma virus-infected cells [1–3]. TGF*β* was shown to transiently confer on normal fibroblasts phenotypic properties of transformed cells, as demonstrated by their acquired ability to grow in soft agar in an anchorage-independent manner [2].

Since then, more than 40 different family members have been identified, including the activin/inhibin subfamily, the bone morphogenetic proteins (BMPs), nodal, myostatin, and the mullerian inhibitory substance (MIS) [4–7]. As for the TGF*β* subfamily, three distinct isoforms have been identified (TGF*β*-1, -2, -3), each encoded by a different gene [4, 8–10]. Of the three different types of TGF*β*, which share around 70% homology within their sequence, TGF*β*-1 has been the most studied isoform and will hereinafter be referred to as TGF*β*.

**Figure 1:**
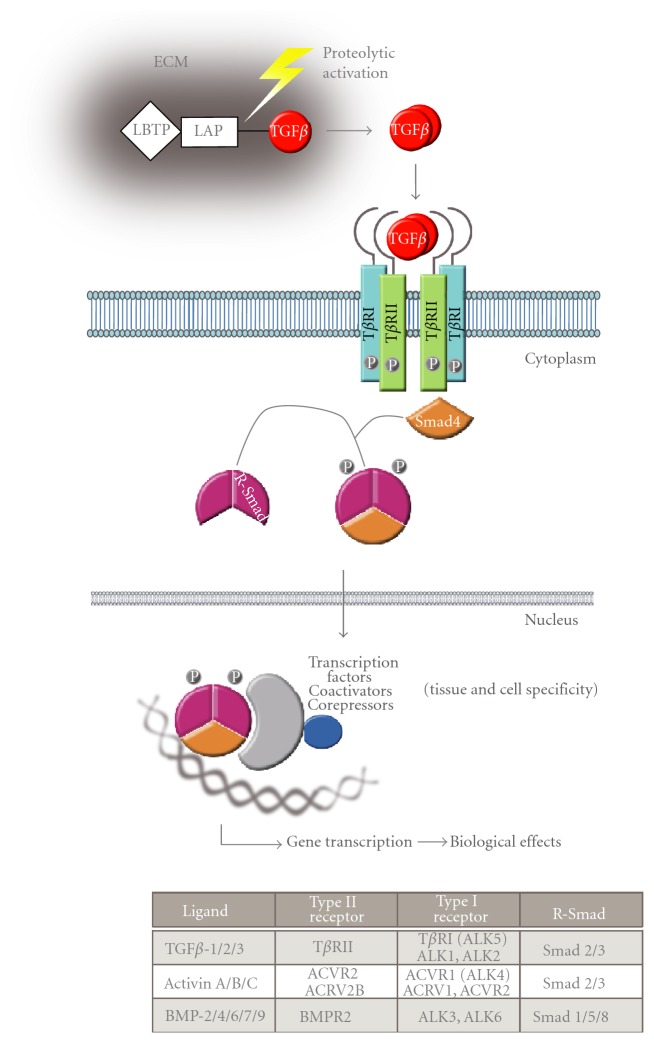
The TGF*β*/Smad canonical signaling pathway. TGF*β* belongs to a superfamily of growth factors that also includes the activins and BMPs. The active TGF*β* ligand is a dimeric molecule composed of two monomers linked by a disulfide bridge and hydrophobic interactions. Each TGF*β* subunit is synthesized as a large inactive precursor molecule bound to accessory proteins (LAP and LTBP). This precursor is stored in the extracellular matrix (ECM) and can be rapidly cleaved and activated by several proteolytic mechanisms to become bioavailable. Signal transduction starts with ligand binding to a complex of specific serine/threonine kinase receptors (type I, type II). The type II receptor is constitutively autophosphorylated and, upon ligand binding, transphosphorylates the juxtamembrane region of the type I receptor. This is followed by phosphorylation and recruitment of the R-Smads to the type I receptor and phospho-R-Smad complex formation with common partner Smad4 in the cytoplasm. The Smad complex is then translocated to the nucleus where it interacts with various transcription factors, coactivators, or corepressors to regulate target gene expression. The table lists the different ligands from the superfamily and their interactions with specific receptors and R-Smad proteins.

The active TGF*β* molecule is a homodimer stabilized by hydrophobic interactions strengthened by a disulfide bond. Each monomer contains *β* strands interlocked by disulfide bonds that form the cysteine knot [11]. This active form of TGF*β* is synthesized from a large inactive precursor molecule, called latent TGF*β*. As shown in Figure 1, latent TGF*β* is composed of a TGF*β* dimer in a noncovalent complex with the TGF*β* propeptide or latency-associated peptide (LAP) that remains bound to TGF*β* after secretion, retaining TGF*β* in an inactive form and the latent TGF*β*-binding protein (LTBP) which is linked to LAP by a disulfide bond [12]. This precursor molecule is stored in the extracellular matrix that acts as a reservoir for TGF*β*. The activation of the TGF*β* precursor is controlled by multiple processes, such as proteolytic enzymatic activity (furins, plasmin, calpain, etc.) but also acid, alkali, and heat-induced proteolysis [12]. Moreover, TGF*β* can be activated by glycosidases, thrombospondin, and by some therapeutic molecules, such as antiestrogens and retinoic acid [13, 14]. The mature TGF*β* is a homodimeric protein composed of two monomeric subunits linked by a single disulfide bond strengthened by some hydrophobic interactions [11].

In 1982, Massagué et al. identified a 60 kDa high-affinity cell surface receptor (type I receptor) for TGF*β* [15]. Subsequently, using affinity cross-linking approaches, other TGF*β* receptors were discovered and identified (type II and type III receptors) [16]. Following identification of its specific receptors, TGF*β* was shown to control and modulate a plethora of biological effects, ranging from cell growth and differentiation, embryogenesis, hormonal synthesis and secretion, immunity, reproduction, bone formation, tissue remodeling and repair, and erythropoiesis, among others [5, 6, 8, 17, 18]. 

TGF*β* and its receptors are widely expressed in all tissues and TGF*β* signal transduction pathways play a major role in human diseases. Indeed, while loss of function has been implicated in hyperproliferative disorders, tumor formation, inflammation, and autoimmune diseases, gain of function leads to immunosuppression and tumor metastasis [6, 9, 19, 20]. Thus, TGF*β* plays a dual role in human cancers, acting both as a tumor suppressor and as a promoter of tumor metastasis. The tumor suppressive effects of TGF*β*, which include inhibition of cell proliferation, induction of apoptosis, and inhibition of cell immortalization, are observed in normal cells and early carcinomas. Conversely, the tumor promoting effects of this growth factor, which include induction of Epithelial-Mesenchymal Transition (EMT), cell adhesion, migration, invasion, chemoattraction, and tumor metastasis, are more specifically observed in aggressive and invasive tumors [6, 21–25]. In addition, as tumors grow and progress, they generally produce and secrete a large amount of autocrine TGF*β* that is then released in the tumor vicinity [26]. These increased TGF*β* levels not only affect the tumor cells themselves but also the surrounding stroma by inhibiting cell adhesion, inducing immunosuppression and angiogenesis, and by promoting the degradation of the extracellular matrix, further contributing to the metastatic process. Thus, the dual role played by TGF*β* and particularly its prometastatic effects make it an attractive target for the development of novel therapies aimed at specifically blocking the pro-metastatic arm of its signaling pathway.

## 2. TGF*β* Signal Transduction

TGF*β* ligands interact with a complex of two transmembrane serine/threonine kinase receptors [5, 8, 27]. Signaling starts with ligand binding to the extracellular domain of the type II TGF*β* receptor (T*β*RII), a constitutively autophosphorylated serine/threonine kinase receptor (Figure 1). Of the three TGF*β* isoforms, TGF*β*2 has the lowest affinity for the type II receptor. As such, TGF*β*2 requires binding to an accessory receptor (type III receptor/betaglycan) first to efficiently bind T*β*RII [28]. TGF*β* binding to T*β*RII is followed by the recruitment of the type I into the complex receptor and its transphosphorylation by the T*β*RII kinase domain. TGF*β* interacts with three distinct type I receptors, including the Activin-Like-Kinase 1 (ALK1), ALK2 or ALK5 [29]. Of note, ALK5 is the predominant form expressed in epithelial cells and is commonly referred as T*β*RI, being the preferred partner for TGF*β*. Given the dimeric nature of mature TGF*β*, the resulting receptor complex is in fact a tetramer, composed of two molecules of T*β*RII associated with two molecules of type I receptor [18]. Phosphorylation of the type I receptors occurs mainly in the juxtamembrane region of the intracellular domain of the receptor, called the GS domain as it is rich in glycine and serine residues [5, 7]. The penultimate residue in the GS domain of the type I receptor, adjacent to the kinase domain, is always a threonine or a glutamine residue. Mutation of this residue to aspartate or glutamate confers elevated kinase activity on the receptor *in vitro* and constitutive signaling activity in the cell, allowing the type I receptor to fully transmit signals in the absence of ligand or type II receptor. The activated type I receptor is the main component of the TGF*β* receptor complex and controls various downstream signaling pathways, including the canonical Smad-dependent pathway [18] as well as non-Smad signaling mechanisms [30].

### 2.1. Smad-Dependent Pathway

 The activated type I receptor recruits and phosphorylates the Smad proteins, the main known effector molecules for these serine kinase receptors. The two receptor-regulated Smads (R-Smads), Smad2 and Smad3, are phosphorylated by the TGF*β* and activin type I receptors (ALK5 and ALK4, resp.) on their C-terminal serine residues (SxS motif). While activin and TGF*β* share the same R-Smad signaling molecules and mostly signal through Smad2 and Smad3, other members of the TGF*β* superfamily, such as the BMPs, signal through distinct R-Smad proteins (Smad1, 5 and 8) following activation of their specific receptors by ligand binding (Figure 1). Once phosphorylated, Smad2 and Smad3 detach from the receptor complex and associate with the common partner Smad4 within the cytoplasm [8, 31–34]. Thus, the activated Smad complex is heterotrimeric, composed of two phospho-Smad2 or phospho-Smad3 moieties with a Smad4 molecule. The Smad complex is then translocated to the nucleus where it acts as a DNA site-specific transcriptional regulator [35]. Nuclear translocation of the Smad complex is regulated by both importin-dependent and -independent mechanisms [36, 37]. Smad proteins recognize the DNA sequence CAGAC, termed the Smad binding element (SBE), as well as some GC rich sequences, but their affinity for DNA is low [38]. In order to achieve a high-affinity DNA binding, the Smads associate with various DNA binding partners [39]. The Smads and associated cofactors bind in concert with their respective cognate recognition sites on DNA, thus ensuring specific selection of the targeted gene promoters and of the TGF*β*-mediated transcription response. These co-factors may be functionally expressed in different cell types thus providing another basis for tissue and cell type-specific functions for TGF*β* ligands [40, 41]. Furthermore, the Smad complex associates with transcriptional coactivators or corepressors, resulting in the induction or repression, respectively, of a given TGF*β*-Smad target gene [42, 43].

### 2.2. Non-Smad Pathways

**Figure 2:**
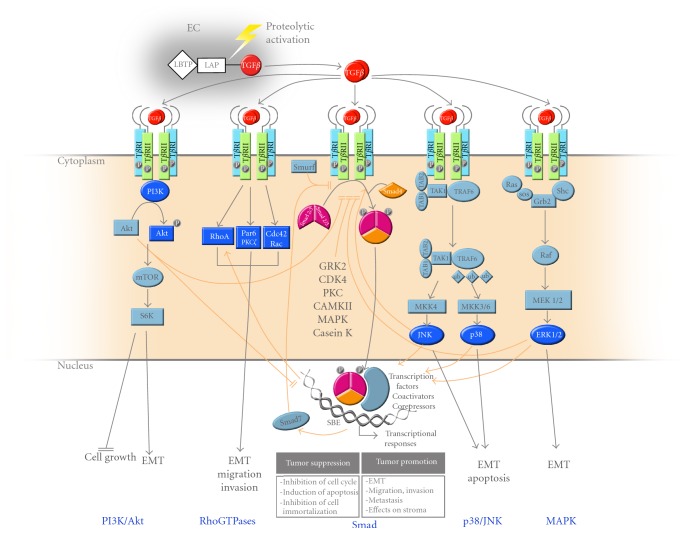
The multiple TGF*β* signaling pathways. The canonical Smad pathway is responsible for most of the TGF*β* biological responses leading to tumor suppression (growth arrest, apoptosis, and prevention of immortalization) and tumor promotion (EMT, migration, invasion, and metastasis). Even though Smads are central to TGF*β* signaling, ligands from this family also signal through other non-Smad pathways. As indicated, TGF*β* can activate the PI3 K/Akt, RhoGTPase, MAPK, and stress-activated kinase (p38/JNK) pathways, leading to various biological effects. Depicted by the orange arrows, these pathways also cross-talk or synergise with the Smad pathway to antagonize or potentiate TGF*β* signaling, respectively. Several Smad inhibitory pathways are also indicated, including TGF*β*-induced gene expression of the inhibitory Smad7, and R-Smad linker phosphorylation by intracellular protein kinases (GRK2, CDK4, PKC, CamKII, MAPK, and Casein kinase).

While the Smad pathway represents the canonical signaling pathway for TGF*β* ligands, other intracellular signaling cascades have been shown to be activated in response to these ligands (Figure 2). In particular, the stress-activated kinases p38 and JNK (*Jun N-terminal Kinase*) have been shown to be induced by TGF*β* ligands and synergize with Smad signaling to lead to apoptosis and epithelial-mesenchymal transition (EMT) [44–49]. The p38 kinase pathway also plays an important role downstream of activin signaling and was shown to be required for activin-mediated cell growth arrest in breast cancer [47] and activin-mediated inhibition of human Pit-1 gene expression in pituitary tumors [49]. TGF*β* can also signal through the mitogen activated protein kinase (MAPK) pathway by activating the extracellular-signal-regulated kinases 1 and 2 (ERK1 and ERK2), further leading to the induction of EMT [45, 50, 51]. Rho GTPases have been shown to relay the TGF*β* signals leading to cytoskeleton reorganization, cell motility, and invasion, through activation of RhoA, Cdc42, and Rac [52, 53]. Finally, TGF*β* was also shown to signal through the mTOR and the phosphoinositide 3-kinase (PI3 K)/Akt pathway to regulate cell growth inhibition [54] and induction of EMT [55, 56]. 

### 2.3. Shutting off TGF*β* Signaling

 Although mechanistically simple, the TGF/Smad signaling cascade is regulated by multiple autoregulatory mechanisms that exist to maintain tightly regulated TGF*β*-induced responses. The first TGF*β*/Smad inhibitory pathway that has been described involves a Smad family member, Smad7. The inhibitory Smad7 functions through a negative feedback loop mechanism to terminate signaling by sterically preventing access of Smad2/3 to the kinase domain of the type I receptor [57, 58]. In addition, Smad7 also recruits protein phosphatases and ubiquitin ligases (Smurf1/2) to the activated TGF*β* receptor, further contributing to the termination of TGF*β* signaling [59–62]. Receptor internalization and receptor downregulation are also important means of regulating TGF*β* signaling [63]. The TGF*β* receptors can be constitutively internalized by clathrin-independent or -dependent mechanisms, through the recruitment of endocytic adaptors like AP-2 and *β*arrestins to the TGF*β* receptor [64, 65]. At the Smad level, nuclear translocated Smad2 and Smad3 can be subject to ubiquitin-proteasome-mediated degradation or dephosphorylation, leading to termination of signaling in both cases [66–69]. Cross-talk from other signaling pathways, such as epidermal growth factor (EGF) and oncogenic Ras signaling, which interfere with the nuclear translocation of the Smads, can further act to negatively regulate Smad-dependent signal transduction [70–72].

While phosphorylation of the C-terminal MH2 domain of the Smad by the type I receptor leads to activation of the R-Smads, phosphorylation of the linker domain by various nonreceptor intracellular kinases inhibits Smad signaling. As shown in Figure 2, such kinases include the MAPK kinases [70, 73], calcium-calmodulin-dependent protein kinase II [74], cyclin-dependent kinase CDK2/4 [75], casein kinase [76], protein kinase C [77], and G protein-coupled receptor kinase 2 (GRK2) [78]. These kinases specifically target the linker region of the R-Smads on multiple distinct serine and threonine residues, leading to termination of Smad signaling. Thus, the linker domain appears as a primary site for negative regulation of Smad signaling. In the case of GRK2, the kinase itself is regulated by TGF*β* signaling and acts in a negative feedback loop [78]. Because GRK2 plays a central role in modulating G-protein coupled receptor signaling, we also found TGF*β*-induced GRK2 expression antagonizes angiotensin II-regulated vascular smooth muscle cell proliferation and migration [79]. GRK2 physically interacts with the MH1 and MH2 domains of the receptor-regulated Smads and phosphorylates their linker region on a specific single serine/threonine residue [78]. GRK2-induced Smad phosphorylation then leads to complete inhibition of TGF*β*-induced Smad activation, nuclear translocation, and target gene expression and inhibits the TGF*β* antiproliferative and pro-apoptotic responses. Thus, GRK2 appears as a novel TGF*β* antagonist that strongly inhibits cell growth arrest and apoptosis in both normal and cancer cells [78]. Interestingly, mutating the GRK2 phosphorylation site within the Smad linker domain to an aspartate residue to mimic a constitutively phosphorylated Smad generates dominant negative Smads that efficiently inhibit TGF*β* responses [80].

## 3. TGF*β* as a Tumor Suppressor

**Figure 3:**
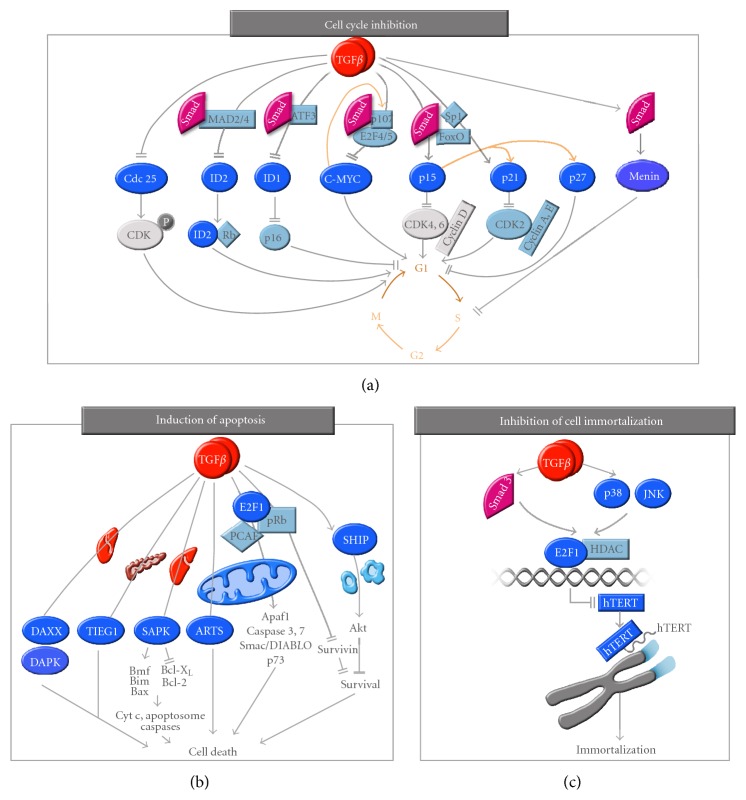
TGF*β* and tumor suppression. (a) *Cell cycle inhibition*. TGF*β* exerts strong cytostatic effects and induces cell cycle arrest in the G1 phase by increasing the expression of the small cyclin-dependent kinase inhibitors p15, p21, and p27. These effects are Smad-dependent but also require the transcription factors Sp1 and FoxO. p15 directly inhibits CDK4/6 and displaces p21 and p27 from their preexisting CDK4/6 complexes allowing them to bind and inhibit CDK2-cyclin A/E complexes (orange arrows). TGF*β*-induced cell cycle arrest also relies on the downregulation of the oncogene c-myc through Smads and repressor E2F4/5. The transcription factors from the ID family are also repressed by TGF*β* through Smads, MAD2/4, and ATF3, further contributing to TGF*β*-mediated cell cycle arrest. Finally, other pathways, potentially more tissue specific, have been described, including upregulation of the tumor suppressor menin in pituitary adenomas, leading to G1 arrest, and downregulation of the tyrosine phosphatase CDC25A in mammary epithelial cells, also leading to G1 arrest. (b) *Induction of apoptosis*. A central pathway in the mediation of the TGF*β* proapoptotic effects involves the E2F1-pRb-P/CAF pathway that leads to gene transcription of multiple TGF*β* proapoptotic target genes in various types of normal and cancer cells. In hematopoietic cells, TGF*β* specifically induces expression of the lipid phosphatase SHIP, which in turn decreases second messenger PIP3 level and blocks Akt-mediated survival pathways, leading to cell death in both B and T lymphocytes. Other tissue specific proapoptotic pathways have been described downstream of TGF*β*, including the TGF*β*-mediated induction of the two proapoptotic proteins DAXX and DAPK in liver cells, the transcription factor TIEG1 in pancreatic cells, and the mitochondrial protein ARTS. TGF*β* also promotes apoptosis in an SAPK-dependent manner by inducing pro-apoptotic target gene expression (Bmf, Bim and Bax) and by repressing antiapoptotic gene expression (Bcl-Xl and Bcl-2), further inducing mitochondrial release of cytochrome C and activation of the apoptosome, leading to caspase-dependent apoptosis in hepatocytes and B-lymphocytes. In colon cancer, TGF*β* was also shown to inhibit expression of the prosurvival protein survivin. (c) *Inhibition of cell immortalization*. TGF*β* also exerts its tumor suppressive effects through inhibition of cell immortalization in normal and cancer cells. This effect is mediated through the Smad, p38, and JNK pathways and requires recruitment of histone deacetylases (HDAC) to the telomerase (hTERT) gene promoter, further leading to inhibition of telomerase expression, and thereby preventing cell immortalization.

The most well-characterized effects of TGF*β* are its tumor suppressor effects in epithelial, endothelial, myeloid, and lymphoid cell types. Expression of the TGF*β* type II receptor (T*β*RII) in breast cancer cells prevents tumor formation [81], while inactivating mutations or overexpression of a dominant negative form of the receptor abolish TGF*β* tumor suppressive effects and increase tumorigenicity [82–84]. Moreover, low expression levels of T*β*RII are correlated with more advanced and aggressive tumor stages, suggesting that the TGF*β* signaling pathway acts as a tumor suppressor in the early stages of tumor development [85]. As shown in Figure 3, TGF*β* exerts strong cytostatic effects on most of its target tissues by inhibiting cell cycle in the G1 phase. In addition, TGF*β* induces apoptosis and prevents cell immortalization in numerous target tissues [4, 24, 48, 86]. 

### 3.1. Cell Cycle Inhibition

Cell cycle progression is controlled by intracellular protein kinases, called cyclin dependent kinases (CDKs). Once activated and associated to their regulatory cyclin subunits, the CDKs induce gene transcription of a number of cell cycle regulators (DNA polymerases, oncogenes, etc.), allowing for cell cycle progression from G1 to S phase. As shown in Figure 3, TGF*β* induces cell cycle arrest in G1 by inducing the expression of small inhibitory molecules, the cyclin-dependent kinase inhibitors (CDKIs) p15^INK4B^ [87] and/or p21^KIP1^ [88], which in turn inhibit specific CDK activity. TGF*β*-induced gene transcription of p15 and p21 is mediated by Smad association with specific transcription factors, such as FoxO forkhead [89] and Sp1 [90, 91]. p15^INK4B^ interacts with either CDK4 or CDK6 or with CDK4-cyclin D or CDK6-cyclin D complexes, while p21^CIP1^ interacts with CDK2-cyclin A or CDK2-cyclin E complexes [92]. TGF*β*-induced p15^INK4B^ expression leads to p15^INK4B^ binding to CDK4 and CDK6, blocking their association with their regulatory cyclins, thereby inhibiting their function and inducing G1 arrest. Moreover, p15^INK4B^ binding to the cyclin D-CDK4/6 complexes also displaces p21^CIP1^ or the related p27^KIP1^ from these complexes, thus allowing these proteins to bind and inactivate CDK2-cyclin A and CDK2-cyclin E complexes [92, 93].

In addition, TGF*β* represses the expression of growth promoting factors such as the oncogene c-MYC [78, 94], and the ID family of helix-loop-helix transcription factors (ID1, ID2, and ID3) [95–97]. These proteins regulate angiogenesis, cell growth, and differentiation and are often upregulated in human cancer [95, 96, 98]. Thus, inhibition of their expression by TGF*β* largely contributes to this growth factor's antiproliferative effect. TGF*β*-mediated c-MYC downregulation is mediated by a transcriptional regulatory complex including Smad3, Smad4, the repressor E2F4/5, and p107 [99] and directly leads to cell growth arrest. Interestingly, expression of the two CDKIs p15^INK4B^ and p21^CIP1^ is normally restrained by the binding of c-MYC and the zinc-finger protein MIZ1, in the proximal region of their promoters [100, 101]. Inhibition of c-MYC expression by TGF*β* thus further contributes to increased expression of p15^INK4B^ and p21^CIP1^ and induction of G1 arrest. The ID proteins interact with the retinoblastoma tumor suppressive protein (pRB) to promote cell proliferation and have been implicated in promoting tumorigenesis [96, 98]. ID1 also delays cellular senescence in primary mammalian cells through inhibition of the cell cycle regulatory protein p16^INK4a^. TGF*β* inhibits ID1 expression in a Smad3-dependent manner through induction of the activating transcription factor-3 (ATF-3), a well-known ID1 repressor [95]. As c-MYC binds the ID2 gene promoter and activates ID2 gene expression, its downregulation by TGF*β* also leads to inhibition of ID2 gene transcription [96, 97]. In ovarian cancer cells, we also found the engulfment protein GULP to act as a key regulator of TGF*β*-mediated growth inhibition [102]. Finally, TGF*β* was also shown to specifically inhibit expression of the tyrosine phosphatase CDC25A in normal mammary epithelial cells, by means of a Smad3/E2F4/5/p130 inhibitory complex [103]. CDC25A normally dephosphorylates an inhibitory site on CDK4 and CDK6. Thus, TGF*β*-mediated inhibition of CDC25A expression allows for sustained CDK4/6 phosphorylation on their inhibitory sites and further induces cell cycle arrest (Figure 3(a)) [103].

Recent work from our laboratory has also revealed a role for the tumor suppressor menin downstream of TGF*β*/Smad signaling in pituitary adenoma cells [5, 104, 105]. Pituitary adenomas are common monoclonal neoplasms accounting for approximately twenty percent of primary intracranial tumors with prolactin-secreting pituitary adenomas (prolactinomas) and are the most common form of pituitary tumors in humans [106, 107]. They are associated with very high levels of the hormone prolactin, exhibit increased tumor growth, and they give rise to severe endocrine disorders, including amenorrhea, infertility issues associated with galactorrhea in females, and impotence in males [106–108]. Multiple endocrine neoplasia type 1 (MEN1) is an autosomal dominant disorder characterized by endocrine tumours of the parathyroid, pancreatic islets, and anterior pituitary, particularly prolactinomas [5, 109]. We found menin to physically interact with Smad3 in somatolactotrope cells and showed that inactivating menin expression antagonizes TGF*β* signaling [104]. We found that menin suppresses TGF*β*-induced transcriptional activity by inhibiting the binding of the Smads to DNA [104]. The role of menin is not restricted to TGF*β* as we also found menin to be required for activin signaling in pituitary cells [110]. Results from our laboratory indicate that activin negatively regulates prolactin gene expression through reduction of Pit-1 expression in a Smad- and menin-dependent manner and that menin is required for activin-induced cell growth inhibition in somatolactotrope cells, highlighting a critical role for activin in mediating pituitary cell growth and Pit-1/prolactin gene expression through the Smads and menin [5, 110].

### 3.2. Induction of Apoptosis

TGF*β* stimulates cell death in various target tissues and these effects have been particularly well documented in the various epithelium, liver, and immune system [111–116]. However, the molecular mechanisms and signaling pathways underlying these pro-apoptotic effects of TGF*β* remain largely uncharacterized. Several apoptotic regulators have been implicated downstream of the TGF*β* signaling pathway, often in a cell- or tissue-specific manner. For instance, in hepatocarcinomas, the Daxx adaptor protein couples the TGF*β* signaling pathway to the cell death machinery through its interaction with the type II TGF*β* receptor (T*β*RII) [117]. Interaction of the Daxx protein to T*β*RII leads to its stabilization and further activation of the JNK and Fas-mediated apoptotic pathways. In liver cancer, TGF*β* also induces gene expression of the death-associated protein kinase DAPK, which promotes cell death, in a Smad-dependent manner, thereby linking the Smad proteins to the mitochondrial proapoptotic processes [118]. The TGF*β*-inducible early-response gene (TIEG1) is a Krüppel-like zinc finger transcription factor that mediates apoptosis in pancreatic epithelial cells [119]. Another mitochondrial protein that has been shown to mediate some of the TGF*β* responses is the septin-like protein ARTS (apoptosis-related protein in the TGF*β* signaling pathway), which can potentiate apoptosis induced by TGF*β*, even in cells resistant to TGF*β*-mediated cell death [120]. The stress-activated protein kinase/c-Jun N-terminal kinase (SAPK/JNK) signaling pathway also plays a critical role in mediating TGF*β*, through Smad interaction with the activator protein AP1 [121, 122]. TGF*β* causes Smad- and the SAPK/p38-dependent transcriptional induction of the pro-apoptotic Bcl-2 family members Bmf and Bim, which in turn activate the pro-apoptotic factor Bax that induces mitochondrial release of cytochrome c and activation of the apoptosome, leading to caspase-dependent apoptosis in hepatocytes and B-lymphocytes [123, 124]. Conversely, the anti-apoptotic proteins Bcl-X_L_ and Bcl-2 have been demonstrated to be down-regulated by TGF*β* in various cell types [125–128]. Each of these signaling events eventually couples TGF*β* to the cell death machinery, leading to changes of expression, localization, and activation of members of the Bcl-2 family and caspases [116]. Interestingly, the pro-apoptotic effects of TGF*β* are particularly strong in immune cells. Work from our laboratory showed that in lymphocytes, both TGF*β* and activin induce the expression of the Src homology 2 domain-containing 5′ inositol phosphatase (SHIP), leading to immune cell death [113]. TGF*β*-induced SHIP expression is Smad-dependent and results in intracellular changes in the pool of phospholipids. Upon TGF*β* stimulation, the increased SHIP expression leads to decreased levels of second messenger PIP3 (Phosphatidyl Inositol triphosphate), further contributing to inhibition of the Akt survival pathway and resulting in cell death in both B and T lymphocytes [113]. TGF*β* also antagonizes survival signaling by inhibiting expression of the prosurvival protein survivin through the physical interaction of Smad3 with Akt, leading to apoptosis in colon cancer [129–131]. In prostate epithelial cells, inhibition of survivin by TGF*β* is Smad-dependent and involves recruitment of a pRb/E2F4 repressive complex to the survivin promoter [132]. Finally, we recently uncovered a central mechanism by which TGF*β* induces apoptosis in both normal and cancer cells of various origins [133]. Indeed, we found TGF*β* to increase expression of the transcription factor E2F1, further leading to the formation and binding of a transcriptionally active E2F1-pRb-P/CAF complex on multiple TGF*β* pro-apoptotic target gene promoters, thereby activating their transcription and highlighting E2F1 as a central mediator of the TGF*β* apoptotic program (Figure 3(b)) [133].

### 3.3. Prevention of Cellular Immortalization

Normal cells are only able to replicate a defined number of times, called the Hayflick limit [134], after which cells enter senescence and die. This limited number of replication cycles is due to the progressive shortening of the ends of the chromosomes, called telomeres, as DNA polymerases fail to completely replicate genetic material at each cell division. As a result, after a number of cell divisions, the length of the telomeres shortens to a critical point, eventually leading to chromosome instability, senescence, and cell death. Interestingly, cancer cells are not subjected to this limitation and thus achieve immortalization, due to the reactivation of an enzymatic program, the telomerase activity. In fact, elevated telomerase activity is so commonly observed in cancer cells that it is being used as a prognostic marker for cancer. Telomerase is a reverse transcriptase that adds telomeric DNA repeats at the end of chromosomes, thereby preventing their shortening. Interestingly, TGF*β* regulates the levels of human telomerase reverse transcriptase (hTERT), the protein component of the telomerase enzyme, by repressing its expression in normal and cancer cells [48, 135, 136]. This TGF*β*-mediated repression of telomerase is Smad3-specific and requires the transcription factor E2F1 as well as the stress-activated kinase and histone deacetylase activities (Figure 3(c)) [48].

In summary, TGF*β* acts as a tumor suppressor, acting through three different signaling arms (cell cycle inhibition, induction of apoptosis, and prevention of cell immortalization) and it is by the combined effects of these three separate signaling axes that TGF*β* exerts its potent tumor suppressive effects in most cell types and tissues.

## 4. Genetic Defects in the TGF*β* Signaling Components and Human Cancer

The role of TGF*β* as a potent tumor suppressor is further highlighted by the fact that many inactivating mutations in TGF*β* receptors and Smad genes have been found to be an underlying cause for human cancer [4, 9, 24, 137]. Multiple genetic and epigenetic alterations of the TGF*β* signaling pathway components have been reported to inhibit TGF*β* tumor suppressive effects, thereby favoring tumor development [137]. These are often found in human cancers of various origin (Table 1) [4, 6, 9, 24, 137] and clearly illustrate the critical role played by the TGF*β* signaling pathway in preventing tumor formation.

### 4.1. Mutations in the TGF*β* Receptor Genes

Mutations in either alleles of the TGF*β* type II receptor (*T βRII*), leading to the formation of truncated or kinase inactive mutant forms of the receptor, are frequently found in colorectal, gastric, biliary, pulmonary, ovarian, esophageal, head and neck cancers, and gliomas [137, 138]. They also occur in other types of tumors, such as those of the endometrium, pancreas, liver, and breast cancers, though with a lower frequency [137]. These inactivating mutations of T*β*RII are more frequently observed in tumors with microsatellite instability, due to mutations in mismatch repair genes. The type I TGF*β* receptor (*TβRI*) also often harbors frameshift and missense mutations in ovarian, breast, esophageal, pancreatic, and head and neck cancers. Epigenetic alterations of the TGF*β* receptor genes, such as promoter hypermethylation or altered/defective expression of the transcription factors that regulate their expression, also lead to decreased receptor expression and inefficient receptor activity [85]. 

### 4.2. Smad Mutations

Like the TGF*β* receptors, the genes coding for the Smad proteins are often mutated or deleted in human cancers [137]. Smad mutations are due to loss of chromosome regions, deletions, frameshift mutations, nonsense, and missense mutations [139]. Of the three domains that compose the Smad molecules, it is within the carboxy-terminal MH2 domain of the Smads that these mutations preferentially occur [137, 140]. These mutations are mostly found in Smad2 and Smad4 and either prevent complex formation with the Smad partners or block activation of Smad-mediated gene transcription [139, 140]. As shown in Table 1, the tumor suppressor Smad4, also known as dpc4 (deleted in pancreatic cancer), is particularly affected by these genetic alterations, being mutated or deleted in no less than half of human pancreatic cancer, where it was originally characterized [141]. Since then, mutations in the Smad4 gene have been characterized in other type of tumors (Table 1) [137]. Mutations in the Smad2 gene also have a relatively high occurrence in lung, liver, and colorectal cancers [137, 142], while the rate of mutation in the Smad3 gene is much lower. In fact, to date there are only few examples of such defects in Smad3 expression, found in some gastric cancers and certain types of leukemia [143].

Finally, inhibition of Smad expression in human tumors can also result from gene amplification of Smad transcriptional repressors. In particular, the two Smad repressors SnoN and Ski are often found activated in in human cancers, highlighting their potent oncogenic properties [144]. Similarly, overexpression of the inhibitory Smad family member, Smad7 has been reported in several types of human cancers, including pancreatic [145], endometrial [146], and thyroid follicular [147] tumors, resulting in inhibition of TGF*β*/Smad signaling.

### 4.3. Mutations/Alterations in the Non-Smad TGF*β* Signaling Pathways

Besides the known mutations in the TGF*β* receptors and canonical Smad pathway, other types of genetic alterations have also been reported to affect TGF*β* signaling and tumor formation. For instance, oncogenic activation of the Ras-Raf-MAPK pathway and c-Jun NH2-terminal kinase in hepatocellular carcinoma has been reported to induce phosphorylation of the Smad3 linker domain by MAPK, further preventing C-terminal phosphorylation of the Smad by the T*β*RI kinase domain and inhibiting TGF*β* cytostatic effects [72]. Moreover, epigenetic alterations of other signaling components can also favor the TGF*β* pro-metastatic effects. Indeed, hypomethylation of the Platelet-Derived Growth Factor *β* (PDGF*β*) gene promotes glioblastoma cell proliferation in response to TGF*β* [148]. Epigenetic downregulation of human disabled homolog 2 (DAB2) also switches TGF*β* from a tumor suppressor to a tumor promoter in head and neck carcinomas [149]. Finally, the Sine oculis homeobox homolog 1 (Six1) homeoprotein was also shown to induce human mammary carcinoma cells to undergo epithelial-mesenchymal transition and metastasis in mice through increasing TGF*β* signaling [150]. 

## 5. TGF*β* as a Prometastatic Factor

As described in the previous sections, the tumor suppressive role of TGF*β* has been well described in multiple target tissues. Interestingly, while TGF*β* acts as a tumor suppressor in normal cells and early carcinoma, its cytostatic effects are often lost during the progression of the disease. Indeed, as indicated above, many different human tumors are either resistant to the TGF*β* cytostatic effects, due to genetic and epigenetic modifications in the TGF*β* signaling components, or become resistant, due to the activation of prooncogenic signaling pathways (MAPK, PI3 K, Ras, c-MYC), which then simply override any growth inhibitory signaling pathways, including TGF*β*/Smad [9, 20, 72, 151]. Meanwhile, other TGF*β* responses prevail, unrelated to the TGF*β* cytostatic effects, which favor tumor progression and metastasis [6, 9, 20, 151]. 

These pro-metastatic effects of TGF*β* were best characterized in breast cancer, the most common form of cancer in women in North America. In 2012, 1.8 million new cases of cancer will be diagnosed in the US and Canada alone, including 250,000 new cases of breast cancer. For 2012 alone, the breast cancer-related death toll is estimated at 45,000 individuals in the US/Canada (http://www.cancer.org/, http://www.cancer.ca/). There are several classifications of breast tumors, depending on the tumor size, the presence of tumor cells outside the primary site either in the lymph nodes or distant metastatic sites, and the growing speed rate of the cancer cells (established from biopsies) [152]. While the rate of remission and overall survival are high in the case of localized primary tumors, they are dramatically lower for metastatic tumors that have propagated to distant sites. The growth of these tumors is independent of hormonal and human epidermal growth factor receptor 2 (HER2) levels. As a result, they usually are insensitive to hormone-based therapies (Tamoxifen, aromatase inhibitors) and therapies targeting the HER2 receptor (Trastuzumab, Herceptin). These aggressive metastatic tumors are responsible for the large majority of breast cancer-related deaths [153]. 

Although typically associated with the TGF*β* cytostatic responses, the Smad proteins are also critical for TGF*β*-mediated tumor metastasis. Indeed, expression of dominant negative Smad3 or expression of a mutant form of the TGF*β* type I receptor that fails to recruit the Smad proteins, in human mammary epithelial cells, significantly diminished their ability to colonize the lungs [154, 155]. Moreover, overexpression of the inhibitory Smad7 impaired mammary carcinoma cell invasion [156]. Finally, gene silencing of the common partner Smad4 in MDA-MB231 invasive breast cancer cells impaired their ability to form osteolytic lesions and the formation of bone metastasis [157, 158].

**Figure 4:**
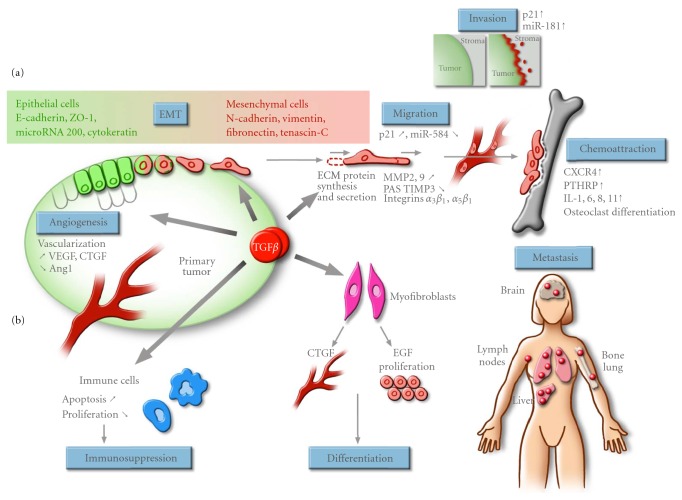
TGF*β* prometastatic effects. Tumor cells synthesize and secrete a significant amount of TGF*β*, which affects both the cancer cell and the stroma. As a result, TGF*β* promotes tumor progression and metastasis by acting directly on the cancer cells themselves and by affecting the stroma and surrounding environment. (a) In cancer cells, TGF*β* promotes the epithelial to mesenchymal transition (EMT) by decreasing cell adhesion and by blocking expression of epithelial proteins (E-Cadherin, ZO-1, etc.) while increasing the expression of mesenchymal proteins (N-Cadherin, vimentin, fibronectin, tenascin-C). TGF*β* also promotes cell migration and invasion through multiple signalling pathways (increased p21 expression, microRNA regulation, increased synthesis and secretion of metalloproteinase expression, activation of RhoGTPases, decreased TIMP3 expression, and regulation of the plasminogen activator system (PAS)). TGF*β* also promotes tumor metastasis by potentiating chemoattraction of the cancer cells to distant organs (bone, lymph node, lung, liver, and brain) and by increasing expression of cytokines (CXCR4, IL-11 and PTHrP) that will promote osteoclast differentiation and the development of osteolytic lesions. (b) TGF*β* affects the stroma and the surrounding environment to varying degrees. TGF*β* induces angiogenesis and stimulates the vascularisation surrounding the tumor by increasing VEGF and CTGF expression in epithelial cells and fibroblasts. Furthermore, TGF*β* also inhibits expression of angiopoetin-1 in fibroblasts, thus increasing permeability of blood vessels associated to the tumor. By inducing hematopoietic cell death, TGF*β* induces local and systemic immunosuppression, preventing the immune cells from infiltrating the tumor and allowing the tumor to escape host immunosurveillance. TGF*β* also promotes myofibroblast differentiation, further promoting tumor growth.

As shown in Figure 4, TGF*β* plays a major role in promoting breast cancer migration, invasion, and metastasis by acting at various levels: (a) on the stroma and neighboring cells surrounding the tumor and (b) directly on the cancer cells themselves. These pro-metastatic responses of TGF*β* include the ability to remodel the surrounding extracellular matrix (ECM), through stimulation of matrix metalloproteinase (MMP) expression and modulation of the plasminogen activation system, resulting in TGF*β*-mediated matrix degradation and, consequently, an increasing release of stored TGF*β* from the ECM that acts as a TGF*β* reservoir [159]. Indeed, in many types of cancer, increased production of TGF*β* correlates with higher tumor grade [159, 160]. Increased TGF*β* expression is observed in breast tumors and correlates with the aggressiveness and advanced stage of the tumor [26]. Interestingly, in mammary carcinoma patients, immunocytochemical analysis revealed that secreted TGF*β* strongly localizes to the advancing edges of the primary tumor and to lymph node metastases [161, 162]. This tumor-derived TGF*β* can exert autocrine effects, that is, effects on the tumor cells themselves, as well as paracrine effects on components of the tumor milieu such as stromal fibroblasts, endothelial cells, and immune cells. Increased secretion of TGF*β* affects and stimulates angiogenesis, contributes to myofibroblast differentiation and causes local and systemic immunosuppression, further contributing to tumor progression and metastasis [9, 20, 137, 151]. As outlined above, cancer cells themselves respond to and are affected by the increased TGF*β* levels, which leads to phenotypic change of the cancer cells from epithelial to mesenchymal (EMT transition), loss of polarity and adhesion, associated with increased migration and invasion cell properties, as well as increased chemoattraction to distant tissues (e.g., bone), thereby favoring and inducing metastatic development [9, 20]. 

Thus, the tumor-permissive effects of TGF*β* provide for a unique therapeutic opportunity in that specifically blocking this signaling network may interrupt mechanisms that are essential for tumor metastasis. However, it is important to note that because of the dual role played by TGF*β*, acting as both tumor suppressor and tumor promoter, elucidation of the molecular events and components leading to both arms of TGF*β* signaling will be critical to further design therapeutic strategies aimed at specifically blocking TGF*β*-mediated tumor metastasis without affecting the tumor suppressive effects of this growth factor.

### 5.1. Paracrine Activity of Tumor-Derived TGF*β*: Effects on the Tumor Microenvironment

#### 5.1.1. Immunosuppression

Elevated TGF*β* levels released from the ECM reservoir exert a profound effect on the immune system. Indeed, as mentioned above, TGF*β* acts as a potent inducer of apoptosis in immune cells. Through up-regulation of the lipid phosphatase SHIP and subsequent inhibition of the PI3kinase/Akt survival pathway, TGF*β* can induce cell death in both B and T lymphocytes [113]. TGF*β* directly targets cytotoxic T cell functions during tumor evasion of immune surveillance by suppressing production of cytolytic factors (pore-forming protein perforin, caspase-activating factors granzymes A and B, and pro-apoptotic cytokines Fas-ligand and interferon *γ*) [163]. TGF*β* also inhibits expression and activity of interleukin-2 and its receptors [164] and blocks T lymphocyte stimulation by dendritic cells during an immune response [165]. TGF*β* inhibits proliferation and differentiation of T lymphocytes, lymphokine-activated killer cells, natural killer cells (NK), neutrophils, macrophages, and B cells [166]. Finally, TGF*β* also decreases tumor cell surface immunogenicity by inhibiting expression of major histocompatibility complex class II antigens, through a Smad3-dependent mechanism [167–169]. These immunosuppressive effects of TGF*β* are best illustrated by the blockade of the TGF*β* signaling cascade through overexpression of a dominant-negative receptor (DN-T*β*RII), which restored both CD8^+^ and CD4^+^ mediated immune response [170]. Thus, the increased concentration of released TGF*β* in the tumor vicinity dramatically contributes to the tumor progression process, as local and systemic immunosuppression induced by TGF*β* allows the tumor to escape host immunosurveillance [4, 6, 137].

#### 5.1.2. Angiogenesis

The process of angiogenesis is essential to tumor growth as it allows blood vessels to deliver nutrients and oxygen to the tumor cells, and allows cancer cells that have detached from the primary tumor to reach and intravasate into the blood system. The TGF*β* signaling pathway is a potent inducer of fibrosis and angiogenesis *in vivo* [171] and promotes chemoattraction of angiogenic cytokine-secreting monocytes [172]. TGF*β* signaling in endothelial cells is rather complex as these cells express two TGF*β* type I receptors (ALK1 and ALK5) [173]. The classic ALK5-mediated pathway leads to Smad2/3 activation, resulting in vessel maturation and angiogenic resolution, while ALK1-mediated signaling antagonizes TGF*β*/ALK5 responses by inducing Smad1/5 and generates transcriptional responses that are linked to angiogenesis [174–177]. The TGF*β* effects in angiogenesis are best illustrated by the multiple germline mutation mice models (ligand, receptors (I, II and III) and Smad1/5) that all lead to vascular and endothelial cell defects [178–185]. Increased expression of TGF*β* correlates with increased microvessel density and with poor prognosis in various tumor types, such as breast cancer and nonsmall cell lung carcinoma [186, 187]. There are many ways in which TGF*β* contributes to the angiogenic process. TGF*β* stimulates the expression of angiogenic factors such as vascular endothelial growth factor (VEGF) and connective-tissue growth factors (CTGF) in epithelial cells and fibroblasts [188, 189]. TGF*β*-induced expression, secretion, and activity of MMPs also contribute to the dissolution of mature vessels around the tumor and the release of endothelial cells from the basement membrane, allowing them to further migrate and invade [190]. Moreover, TGF*β* represses expression of angiopoietin-1, a critical factor in maintaining vessel integrity, in fibroblasts thereby contributing to the permeable properties of tumor-associated blood vessels [191].

#### 5.1.3. Myofibroblast Generation

Many recent studies have focused on the emerging role of tumor-stroma interactions, which are essential for supporting tumor progression. Myofibroblasts, also known as cancer-associated fibroblasts (CAFs), are mesenchymal cells harboring characteristics from both fibroblasts and smooth muscle cells [192]. These cells can secrete numerous cytokines, growth factors, and ECM components and have the ability to substantially promote tumorigenesis and their appearance precedes the invasive stage of cancer. In a coimplantation breast tumor xenograft model, resident human mammary fibroblasts progressively converted into CAF myofibroblasts during the course of tumor progression [193]. During this process, they displayed increased autocrine signaling loops, mediated by TGF*β* and SDF-1 cytokines, which acted in both auto-stimulatory and cross-communicating fashions. These autocrine-signaling loops initiated and maintained the differentiation of fibroblasts into myofibroblasts and the concurrent tumor-promoting phenotype [193]. TGF*β* also significantly increased the percent of myofibroblasts and invasion rate in CAF cultures [194] and increased the production and secretion of urokinase-type plasminogen activator (uPA) by human breast myofibroblasts [195]. Thus, TGF*β* induces the generation and maturation of myofibroblasts from precursor fibroblasts which, in turn, stimulate invasion of the tumor cells through secretion of proliferative, proinvasive and proangiogenic factors.

As summarized in Figure 4, TGF*β* plays an important role in promoting tumor growth and development as well as tumor metastasis by allowing tumor cells to survive, detach and migrate away from the primary tumor to invade the surrounding tumor environment and metastasize to distant organs.

### 5.2. Autocrine Activity of Tumor-Derived TGF*β*: Effects on the Tumor Cells

The increased TGF*β* levels produced by the tumor cells also contribute to the formation of a favorable microenvironment for tumor growth and spread by acting directly on the tumor cells themselves. Tumor-produced TGF*β* stimulates EMT, cell migration, and invasion and promotes chemoattraction of the tumor cell towards distant organs (Figure 4).

#### 5.2.1. Epithelial to Mesenchymal Transition

The EMT process characterizes the differentiation of highly organized and tightly connected networks of epithelial cells into disorganized and mobile mesenchymal cells with stem cell-like properties. The EMT process involves a loss of cell-to-cell contact and the acquisition of fibroblastic characteristics by the epithelial cells as well as the acquisition of migratory and invasive properties by the cancer cells [196, 197]. EMT is a naturally occurring process that takes place during embryogenesis and development. EMT drives and governs morphogenesis by inducing the differentiation of the epithelium into mesenchymal cell types to generate the different embryonic territories. The EMT process is characterized by the dissolution of epithelial tight junctions and basolateral adherens junctions, resulting in the loss of epithelial cell polarity. This is best exemplified by the loss of epithelial gene expression (E-cadherin, ZO-1, occludin, claudin, cytokeratins 8, 18, and 19, desmoplakin) and the induction of more mesenchymal markers (N-Cadherin, vimentin, fibronectin, tenascin-C, and vitronectin). During EMT, the actin cytoskeleton is also reorganized from a cortical adherens-associated location into actin stress fibers anchored to focal adhesion complexes that contribute to the formation of filopodia and promote cell migration. EMT also contributes to cancer cell invasion and dissemination. Indeed, down-regulation of E-cadherin allows for the release of *β*-catenin leading to increased expression of c-MYC, cyclin D1, and MMP7, thereby promoting the invasive behavior of the cells. Moreover, during EMT, increased secretion of extracellular proteases and reduced expression of ECM proteins further contribute to cancer cell invasion. EMT is under the control of several key transcription factors that regulate expression of mesenchymal markers and repression of epithelial genes. These include the zinc-finger proteins Snail and Slug, the basic helix-loop-helix factor Twist, the zinc-finger/homeodomain proteins ZEB-1 and -2, as well as the forkhead factor FoxC3 [4], which are all regulated and under the control of TGF*β* [198].

The role of TGF*β* in EMT has been relatively well characterized. TGF*β* induces reversible EMT in both normal and cancer contexts [199, 200]. Reciprocally, blocking TGF*β* signaling by overexpression of a dominant-negative T*β*RII efficiently prevents skin squamous cancer cells from undergoing EMT *in vivo *[201]. Interestingly, the tumor cells located at the invasion front, which contain high levels of TGF*β*, show enhanced EMT features. The canonical Smad pathway plays a central role in mediating the TGF*β*-induced EMT effects. Smad-dependent activation of transcription leads to expression of the EMT regulatory factors, such as Snail, Slug, ZEB-2, and Twist through induction of the expression of high-mobility group A2 (HMGA2) protein [198], resulting in repression of E-cadherin expression [202] and dissociation of desmosomes [203]. Phosphorylation of the cell polarity protein Par6 by T*β*RII also leads to the dissolution of cell junction complexes [204]. While Smad-dependent TGF*β*-induced EMT is enhanced by Ras signaling [205], other Smad-independent TGF*β* downstream signaling pathways, including the Ras/PI3 K [206–208], RhoA [53], mTOR [56, 209], Erk MAPK [50, 210–212], and p38 stress-activated kinase [44] pathways also contribute to TGF*β*-induced EMT.

MicroRNAs (small noncoding RNAs) also play an important role in the regulation and maintenance of EMT downstream of TGF*β* [6, 213]. MicroRNAs (miRNAs) have eluded researchers for decades, stealthily regulating many of the major biological processes in eukaryotic cells. miRNAs regulate gene expression posttranscriptionally by guiding the RNA-induced silencing complex (RISC) to their cognate site of the 3′untranslated region (3′UTR) of the target mRNA. While miRNAs represent only 1% of all human genes, over a third of the transcriptome is regulated by these miRNAs [214]. Individual miRNAs can regulate hundreds of genes directly and thousands indirectly [215, 216]. By controlling and regulating the expression of so many genes, it clearly became apparent that miRNAs play a central and critical role in the pathogenesis of human diseases, including cancer [217–222]. Moreover, about half of the miRNA encoding genes are located in chromosomal regions that are being altered during tumorigenesis [223]. Numerous miRNA signatures have been characterized in human cancers and implicated in the tumorigenic process [6, 224–228]. While some miRNAs exert their effects as classical oncogenes or tumor suppressors [229], others act in the advanced stages of the disease by promoting cancer progression and tumor metastasis [230–233]. Many of these miRNAs regulate TGF*β*-mediated tumor metastasis [6]. As an example, TGF*β* represses expression of miR-200 leading to increased levels of the miR-200 target, ZEB2/SIP1 [213]. As ZEB2/SIP1 acts as the main repressor of E-Cadherin expression, TGF*β*-mediated down-regulation of miR-200 leads to decreased E-cadherin levels and EMT in breast [234], pancreatic [235], and colorectal cancer [236]. In turn, ZEB2/SIP1 targets TGF*β* and miR-200 transcription in a feedforward loop which stabilizes EMT [236].

By inducing EMT and modifying the cell phenotype, TGF*β* alters cell-to-cell contact and communication as well as the adhesive, migratory and invasive properties of the tumor cells (Figure 4).

#### 5.2.2. Cell Adhesion

TGF*β*-induced EMT leads to changes in the expression profiles of adhesion molecules that profoundly diminish cell-to-cell and cell-to-substrate adhesion. These inhibitory effects on cell adhesion promote the detachment of the cancer cells from the primary tumor and their dissemination throughout the stroma. For instance, in the skin, melanocytes are tightly connected to keratinocytes through surface expression of E-cadherin. In melanoma, TGF*β*-induced EMT leads to downregulation of E-cadherin and alters the communication between keratinocytes and melanocytes and further allows melanoma cells to attach and communicate with fibroblasts from the stroma and endothelial cells, thereby favoring their propagation throughout the derma. In osteosarcomas, TGF*β* inhibits cell adhesion to the substrate laminin, by down-regulating expression of the laminin receptor, *α*_3_ *β*_1_ integrin [237]. Interestingly, TGF*β* specifically inhibits cell interaction with laminin, as receptors for other substrates, such as collagen (*α*_2_ *β*_1_ integrin) and fibronectin (*α*_5_ *β*_1_ integrin) are not affected by TGF*β* [237].

#### 5.2.3. Cell Migration

A direct consequence of EMT is the acquisition of migratory and invasive properties by the cancer cells [238]. As mentioned above, TGF*β* regulates the expression of several transcription factors such as HMGA2, Snail, Slug, and Twist during the EMT process. Expression of HMGA2, Snail or Twist alone can induce EMT and increase cell migration [238]. Expression of a dominant negative T*β*RII prevents TGF*β*-induced EMT and blocks migration [239]. Overexpression of constitutively active T*β*RI restores cellular motility through the activation of PI3 K/Akt and MAPK pathways. In order to migrate, cells generate lamellipodia protrusions at the front end while retracting the trailing end. These events are coordinated by Rho-family GTPases which are themselves activated by TGF*β* [240]. Although the induction of Rho signaling by TGF*β* is not fully understood, it has recently been shown that RhoA activator is up-regulated by TGF*β* in a Smad4-dependent manner [240]. While TGF*β* exerts an important role in breast cancer progression as a pro-metastatic factor, notably through enhancement of cell migration, it is becoming clear that microRNAs also play a crucial role in the mediation of these effects [6]. For instance, miR-155 (which is regulated by TGF*β* targets RhoA, thus directly contributing to epithelial plasticity [241]. Interestingly, we recently found TGF*β*-mediated regulation of several microRNAs to be critical for TGF*β*-induced cell migration [242, 243]. In particular, our results highlight a novel signaling route whereby TGF*β* silences expression of the microRNA miR-584, further leading to actin re-arrangement and breast cancer cell migration [242]. TGF*β* down-regulation of miR-584 in breast cancer cells leads to increased expression of its downstream target, the actin-binding protein PHACTR1, resulting in enhanced cellular migration (Figure 4). Accordingly, over-expressing miR-584 or knocking down expression of the target PHACTR1 resulted in a drastic reorganization of the actin cytoskeleton and impaired TGF*β*-induced cell migration [242]. 

#### 5.2.4. Cell Invasion

In addition to its promigratory role, TGF*β* also contributes to the ECM remodeling and invasiveness of the cells by increasing the expression of metalloproteinases and the generation of plasmin which, in turn, contributes to the release of stored TGF*β* from the ECM, further increasing cell invasion [244]. The increased TGF*β* levels allow cancer cells to cross through the ECM to reach distant metastatic sites. In invasive hepatocellular carcinomas, TGF*β* induces transcriptional expression of *α*3*β*1-integrin, a key player in basement membrane invasion [245, 246]. Moreover, TGF*β* has been shown to inhibit expression of tissue inhibitor of metalloproteinase 3, TIMP3, further contributing to hepatocellular carcinoma cell invasion [247]. Though often associated together, TGF*β*-induced EMT can be dissociated from TGF*β*-induced invasion and metastasis. Indeed, using a skin carcinogenesis mouse model, it was found that TGF*β*-mediated EMT requires a functional TGF*β* type II receptor (T*β*RII), whereas TGF*β*-mediated tumor invasion is associated with reduced T*β*RII signaling in tumor epithelia [248]. As outlined above, microRNAs are important regulators of the metastatic process. We and others have demonstrated the mir-181 family of microRNAs to be up-regulated by TGF*β* and activin, a closely related TGF*β* family member [243, 247, 249]. In hepatocellular carcinoma, TGF*β*-induced miR-181 targets TIMP3 for degradation, thereby increasing invasiveness of the cells [247]. We also found miR-181 to be a downstream regulator of activin/TGF*β*-induced cellular migration and invasion in breast cancer (Figure 4). As a critical regulator of tumor cell migration and invasion and breast cancer progression *in vitro*, miR-181 could potentially be an important therapeutic target [243].

Finally, a recent study from our laboratory identified p21Cip^1^ (p21), a member of the core cell cycle machinery, as a key regulator of TGF*β*-mediated breast cancer cell migration and invasion [250] (Figure 4). We found p21 expression to correlate with poor overall and distant metastasis-free survival in breast cancer patients. Furthermore, using *in vivo* xenograft animal models, we found p21 to be essential for local tumor invasion [250]. p21 interacts with Smad3 and the acetyltransferase P/CAF to regulate Smad acetylation and transcriptional activity, as well as gene transcription of downstream TGF*β*-induced pro-metastatic genes [250, 251]. Our data also showed a significant association between TGF*β*/Smad3 signaling, p21, and P/CAF expression with lymph node positivity, making them potential useful prognosis markers for lymph node metastasis. Together these findings highlight an important role for the p21-P/CAF-Smad3 signaling axis in promoting breast cancer cell migration and invasion at the transcriptional level, and support the notion of a direct oncogenic role for p21 in the progression of breast cancer to a metastatic disease [250].

#### 5.2.5. Contribution to Distant Metastasis and Chemoattraction

While TGF*β* directly contributes to local invasion, this is only the first event in a multistep process which will eventually lead to the formation and establishment of secondary tumors [252]. As shown in Figure 4, TGF*β* also contributes to the establishment of metastasis by contributing to the growth of these secondary lesions [4]. Tumor cells that have migrated through and invaded the matrix can penetrate blood vessels, through a mechanism called intravasation. Once in the circulation, tumor cells will then disseminate to distant sites and organs by exiting blood vessels (extravasation) and form new colonies in a new favorable distant microenvironment. TGF*β* stimulates the secretion of osteolytic cytokines, which further contribute to the metastatic process by digesting the bone matrix [253]. These events are Smad-dependent as the formation of osteolytic lesions in mice by breast cancer, melanoma, and renal carcinoma cells can be blocked by overexpressing the inhibitory Smad7 or a dominant negative TGF*β* receptor [254–256]. TGF*β* stimulates the secretion of the parathyroid hormone related protein (PTHrP) which promotes the differentiation of osteoclast precursors and bone resorption [253] and induces expression of the bone homing receptor C-X-C chemokine receptor type 4 (CXCR4) [257]. Association of the stroma-derived factor-1 (SDF-1) ligand to its receptor CXCR4 promotes chemoattraction of the breast cancer cells to the bone secondary sites [4, 258]. TGF*β* also induces the expression of the interleukin proteins IL-1, IL-6, IL-11, and connective tissue growth factor (CTGF), leading to osteoclastic differentiation and angiogenesis, and further contributing to bone resorption and the formation of osteolytic lesions [95, 158, 259–261]. TGF*β*-mediated IL-11 and PTHrP by the cancer cells leads to increased expression of receptor activator of nuclear factor kappa-B ligand (RANKL) at the osteoclast cell surface, further enhancing progenitor cell differentiation into osteoclasts and bone demineralization [159, 160]. In addition to the development of bone osteolytic lesions, TGF*β* also contributes to the development of metastases by directing cells to specific tissues and by enhancing the extravasation of breast cancer cells into the lung parenchyma [262], by inducing expression of cyclooxygenase-2 (COX2), epidermal growth factor receptor (EGFR), and angiopoietin-like 4 (ANGPTL4), and promoting development of lung metastasis [263]. Some of these genes (*COX2 *and *EGFR) *have also been associated with brain metastasis [264]. 

#### 5.2.6. Antagonizing Suppressor of Metastasis Pathways in Breast Cancer

Mammary gland growth and differentiation is a complex process regulated by steroids, polypeptide hormones, and growth factors, among which TGF*β* and the hormone prolactin play major roles. While prolactin is required for lobuloalveolar formation and functional differentiation of mammary epithelial cells, TGF*β* exerts an opposite effect, inducing apoptosis during mammary gland involution and inhibiting milk protein expression [265, 266]. Prolactin signaling is mediated by the interaction of its specific receptor with the intracellular tyrosine kinase Jak2 [267–273]. Once phosphorylated, tyrosine residues on both Jak2 and the prolactin receptor create docking sites for the recruitment and activation of the transcription factor Stat5 which will then activate gene transcription of target genes such as those encoding milk proteins and cell growth regulators [274–276]. The importance of Stat5 in mammary gland development is further highlighted by the Stat5a knockout mouse which show no lobuloalveolar development during pregnancy and a complete absence of lactation [277]. Interestingly, an elegant study from the Ali laboratory, revealed prolactin to also act as a suppressor of metastasis in breast cancer [278]. Indeed, prolactin and Jak2 were shown to play a critical role in regulating epithelial-mesenchymal transition. Activation of the prolactin/Jak2 signaling pathway in mesenchymal-like breast cancer cells suppressed their mesenchymal properties and reduced their invasive behavior while blocking prolactin autocrine function in epithelial-like breast cancer cells induced mesenchymal-like phenotypic changes and enhanced their invasive capacity [278]. Interestingly, blocking prolactin signaling led to activation of the two major prometastatic pathways, the mitogen-activated protein kinase and the TGF*β*/Smad signaling pathways, highlighting prolactin as a critical regulator of epithelial plasticity and defining a new role for prolactin as an invasion suppressor hormone in breast cancer [278]. TGF*β* is expressed in each phase of postnatal mammary gland development [279] and all three isoforms of TGF*β* are up-regulated during mammary gland involution [265, 280, 281]. TGF*β* inhibits alveolar formation, milk protein synthesis and induces apoptosis during involution of the mammary gland [282–284], suggesting that TGF*β* signaling may also antagonize prolactin-induced signals in mammary cells [285, 286]. In a recent study, we identified a novel antagonistic crosstalk mechanism by which TGF*β*/Smad signaling inhibits prolactin signaling and Stat5-mediated gene transcription and mammary epithelial cell differentiation, by preventing Stat5 binding to its coactivator CBP [287]. These studies indicate that the prolactin and TGF*β* signaling cascades oppose their effects not only to regulate differentiation of mammary epithelial cells and lactation but also to modulate tumor formation and breast cancer metastasis [278, 287]. 

## 6. Targeting TGF*β* in Cancer Therapy

**Figure 5:**
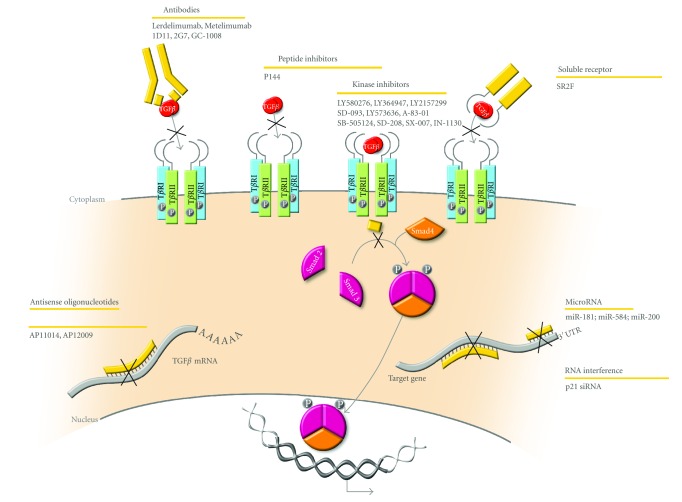
TGF*β* antagonists and inhibitors. Blocking the TGF*β* signaling pathway provides for a unique therapeutic opportunity against tumor metastasis. As such, several approaches to develop new therapeutic tools that would interfere with TGF*β* signaling have been undertaken in recent years. Blocking antibodies, peptide inhibitors, kinase inhibitors, soluble receptors, and antisense oligonucleotides are all being tested, some of which are at different phases of clinical trials. In future, more specific approaches may involve targeting specific microRNAs or making use of RNA interference approaches to block expression of specific downstream TGF*β* pro-metastatic targets (e.g., p21).

As outlined in this paper, TGF*β* tumor suppressive effects are often lost in aggressive tumors, while tumor promoting and pro-invasive responses remain and prevail, leading to the development of distant metastases. Moreover, since TGF*β* expression is increased in many cancers and correlates with the stage of the tumor [159, 160], blocking the TGF*β* signaling pathway may provide for a unique therapeutic opportunity against tumor metastasis. As such, several approaches to develop new therapeutic tools that would interfere with the TGF*β* pathway have been undertaken in recent years (Figure 5) [6, 159, 160, 288–291]. 

### 6.1. Preventing Ligand-Receptor Interaction Using Blocking Monoclonal Antibodies, Soluble Receptors, and Peptide Inhibitors

Blocking antibodies against specific TGF*β* isoforms, such as TGF*β*-1 (Metelimumab or CAT-192; Cambridge Antibody Technology and Genzyme) [289] and TGF*β*-2 (Lerdelimumab or CAT-152 or Trabio; Cambridge Antibody Technology) [292–294] have been developed and tested. However, both proved unsuccessful in clinical trials and were subsequently discontinued [295–298] (reviewed in [6]). As all TGF*β* isoforms influence tumors, pan-TGF*β* antibodies are also being developed, as they may prove more efficient than isoform-specific antibodies [6, 288, 291, 299–302]. Targeting TGF*β* signaling with soluble receptors has also been investigated, using exogenous expression of a soluble T*β*RII [303, 304], soluble recombinant T*β*RIII (betaglycan) [305], decorin, a proteoglycan induced by TGF*β* [306–308] or even a chimeric soluble receptor was constructed by fusing the extracellular domain of T*β*RII to the Fc regions of human immunoglobulin IgG1 (Fc:T*β*RII or SR2F) [307, 308]. Synthetic short peptides derived from TGF*β* receptors that block TGF*β* binding to its receptors are also an interesting avenue. One such promising candidate, P144 (DigNA Biotech), inhibits TGF*β* signaling and collagen type I synthesis in cardiac fibroblasts and potentially prevents myocardial fibrosis in hypertensive rats [309]. 

### 6.2. Blocking TGF*β* Production at the Translational Level, Using Antisense Oligonucleotides

 Antisense oligonucleotides (ASO) are 13–25-nt single-stranded nucleic acids, chemically modified or not, that are complementary to the target mRNA [6, 310, 311]. The compound AP 11014, currently in advanced preclinical studies, specifically targets the TGF*β*-1 mRNA and has been shown to significantly reduce TGF*β*-1 secretion in multiple cancer cell lines, impeding TGF-*β*1-induced immunosuppression [6, 312, 313]. Another compound, known as AP 12009 (or Trabedersen; Antisense Pharma), was designed to target the TGF*β*-2 mRNA and showed some efficacy in pancreatic cancer and glioblastoma [6, 302, 314–317].

### 6.3. Blocking the Downstream Receptor-Mediated Signaling Cascade, by Interfering with the Receptor Kinase Activity, Using Small-Molecule Inhibitors

The development of TGF*β* receptor kinase inhibitors has primarily focused on T*β*RI, due to extensive knowledge of the effect of this receptor on Smad phosphorylation and since targeting T*β*RI would not disrupt potential T*β*RI-independent pathways initiated by T*β*RII [23, 296]. Multiple T*β*RI kinase inhibitor compounds have been developed and tested and showed various levels of efficacy (reviewed in [6]). Some were tested in xenograft models in breast and non-small cell lung cancers and showed tumor growth delay *in vivo *[318]. Oral administration of LY2157299 with advanced malignancies was determined to be safe and well tolerated in a phase I clinical trial [319]. Moreover, SD-093 strongly inhibits the TGF*β*-induced motility and invasiveness of pancreatic carcinoma cells [320] and TGF*β*-induced EMT in mammary epithelial cells [321, 322]. SD-208 and SX-007 efficiently inhibit TGF*β*-induced migration and invasion and prolonged survival in murine glioma tumors [323, 324]. SB-431542, a selective inhibitor of Smad3 phosphorylation by T*β*RI, inhibits TGF*β*-induced fibronectin and type I collagen synthesis in renal epithelial carcinoma cells [325]. LY2109761 inhibits both Smad-dependent and -independent TGF*β* responses and attenuates TGF*β*-induced cell migration, invasion, and tumorigenicity in colon adenocarcinoma [326] and decreases liver metastases and prolonged survival in a murine pancreatic cancer model [327]. 

### 6.4. Importance of the Specificity of Blocking TGF*β* Signaling

One of the main concerns in targeted therapy is the off-target effects. Because TGF*β* exerts a dual role in cancer, targeted therapy to block TGF*β* signaling raises a major concern. Indeed, blocking the TGF*β* pro-metastatic effects will only be beneficial if the therapy does not affect the tumor suppressive arm of TGF*β* signaling. In a Neu-driven breast cancer model, constitutively active T*β*RI increased the latency of the primary mammary tumor but also increased pulmonary metastasis whereas a dominant negative T*β*RII decreased the latency of the primary tumor but also significantly decreased the number of lung metastases [262]. This finding indicates that although TGF*β* acts as a tumor suppressor on the primary tumor, it may act on the ability of the breast cancer cells to extravasate from lung vessels to the parenchyma. Although loss of T*β*RII correlates with poor prognosis in esophageal cancer [328] and renal carcinoma [329], it also correlates with better survival rate in colon cancer [330] and gastric cancer [331], clearly indicating that the role of T*β*RII in carcinogenesis may be stage and tissue specific. 

Overall it seems that the beneficial effects of TGF*β* could be context dependent. These potential risks give rise to the necessity to identify patients in which the pro-metastatic arm of the TGF*β* signaling pathway is predominant. Assessing the levels of TGF*β* in the serum or the tumor has been studied as a tool for screening patients, showing a correlation between high serum levels and tumor progression and metastasis [302]. Many efforts have been made to target TGF*β* in cancer due to its crucial role in cancer progression. Several strategies have been developed and some molecules have shown encouraging and promising results. However, these strategies still have very important challenges to overcome. Alternatively, new strategies aimed at specifically targeting the pro-metastatic arm of the TGF*β* signaling pathway may prove more useful and safer. For this, identifying the downstream signaling components and elucidating the molecular mechanisms by which this growth factor promotes cell migration, invasion, EMT, and metastasis will be critical for establishing successful specific therapies. For instance, RNA interference approaches specifically targeting intracellular downstream molecules relaying these tumor promoting effects of TGF*β* could prove useful. We recently found the cell cycle regulator, p21 to play a central role in the mediation of TGF*β*-mediated local tumor cell invasion [250]. Using *in vitro* approaches and *in vivo* xenograft animal models, we found that blocking p21 expression with specific shRNAs and siRNAs could significantly alter the TGF*β* tumor promoting effects, without affecting cell growth or tumor formation [250]. Thus, designing therapeutic strategies aiming at knocking down p21 expression in breast cancer patients may prove useful to prevent or circumvent the metastatic disease in this tissue (Figure 5).

Another important parameter to consider relates to the tissue specificity of the therapy. Indeed, while the TGF*β* pro-metastatic effects have been relatively well characterized in breast cancer, the role of TGF*β* in tumor cell invasion and metastasis in other tissues remain elusive. For instance, in melanoma, which is the leading cause of death due to cancer in young adults from 25 to 30 years old [332], the TGF*β* effects on tumor progression remain unclear. While some reports suggested that TGF*β* signaling could promote the metastatic potential of the cells [150, 217], other studies, including recent work from our laboratory, indicate that TGF*β* potently inhibits cell migration and invasion of melanoma cells issued from various patients with different clinical backgrounds [333, 334]. Thus, in this particular context, clinical strategies aiming at mimicking the TGF*β* antiproliferative, antimigratory and anti-invasive effects may prove beneficial for the treatment of melanomas at different stages of their progression, including primary and metastatic tumors. 

## 7. Conclusion

**Figure 6:**
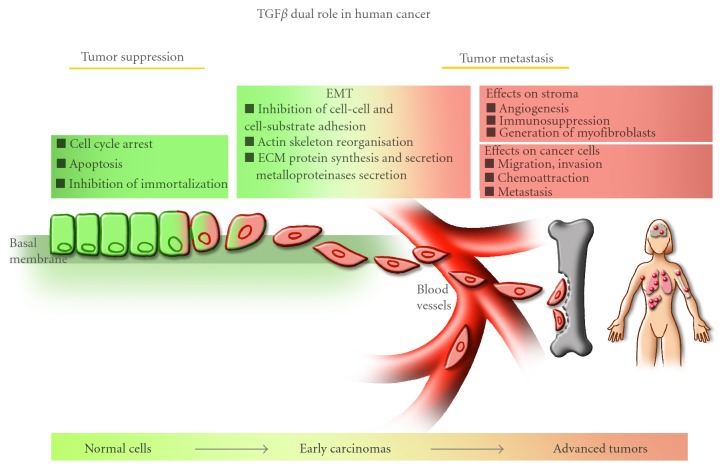
The dual role of TGF*β* in human cancer. In summary, TGF*β* acts as a tumor suppressor in normal cells and early carcinomas, while it promotes tumor metastasis in more advance stages of cancer. The tumor suppressive effects of TGF*β* include cell cycle arrest, apoptosis and prevention of cell immortalization. TGF*β* also induces EMT, marked by a decrease in cell-cell and cell-substrate contact, a reorganization of the actin cytoskeleton, as well as an increase in metalloproteinase synthesis and secretion. In human cancers, inactivating mutations in the TGF*β* signaling components or activating mutations in oncogenic signaling pathways are often observed and provide an underlying basis for tumor development. These mutations attenuate the TGF*β* tumor suppressive effects but do not affect its tumor promoting effects on cancer cells and on the surrounding environment, including EMT, cell migration and invasion, angiogenesis, immunosuppression, myofibroblast generation, chemoattraction, and tumor metastasis, further promoting TGF*β*-induced tumor progression to secondary distant sites.

TGF*β* plays a major role in regulating cancer formation and progression. While acting as a tumor suppressor in normal cells and early carcinomas, TGF*β* switches roles to in fact promote tumor progression in more advanced invasive cancers (Figure 6). Understanding and elucidating the intracellular and molecular mechanisms that trigger the TGF*β* tumorigenic effects will thus be critical for the development of novel anticancer therapies based on the use of TGF*β* antagonists. Combined research from academia and industry has led to the development of such new therapeutic tools, some of which have demonstrated promising results. Although the available TGF*β* antagonists tested so far have shown some relative efficacy in different types of cancer, their use may also be limited. Indeed, even though several antagonists are currently being tested in clinical trials, their long-term efficiency and potential adverse side effects remain to be determined. In particular, it is difficult to predict whether blocking all TGF*β* effects, as with the current strategies, will allow for sufficient blockage of the pro-metastatic arm of the TGF*β* pathway without affecting the tumor suppressive arm, thereby giving rise to spontaneous tumors elsewhere in the organism. In that respect, it will be vitally important to focus efforts on the development of novel strategies aimed at specifically manipulating the downstream signaling components of the TGF*β* tumor promoting effects, as they may prove more effective and safer in the long run.

## Figures and Tables

**Table 1:** Mutations and deletions in the TGF*β* signaling pathway. While expression of TGF*β* itself is often increased in human tumors, expression of the genes encoding various components of the TGF*β* signaling cascade (receptor type I and type II, Smad2, Smad3, and Smad4) are often mutated or deleted in human cancer. Occurrence of mutation and deletion and incidence rates in different human cancers are indicated in percentages. Loss of heterozygosity (LH) is also indicated.

Molecules	Cancers
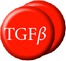	*Increased expression:* breast (68%), lung (48%), pancreas (47%), esophagus (37%), stomach (23%), colon, prostate.

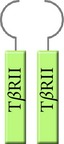	*Mutations/deletions:* colon (28%), ovary (25%), head and neck carcinoma (21%), stomach (15%), breast (12%), lung, endometrium, liver, uterus, biliary track, glyomas.

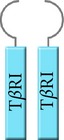	*Mutations/deletions:* ovary (30%), head and neck carcinoma (17%, LH 53%), bladder (LH 31%), prostate (25%), breast (6%), biliary track.

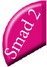	*Mutations/deletions:* colon (8%), uterus (8%), liver, lung.

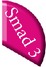	*Mutations/deletions:* lymphoblastic leukemia, stomach.

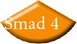	*Mutations/deletions:* pancreas (50%, LH 90%, deletion 30%), colon (LH 60%), stomach (LH 60%), lung (LH 56%), breast (12%, LH 30%), head and neck carcinoma (LH 40%), prostate (LH 30%), biliary track (16%), uterus (4%), bladder, oesophagus, kidney, liver, ovary
